# Oligomeric Coiled-Coil Adhesin YadA Is a Double-Edged Sword

**DOI:** 10.1371/journal.pone.0015159

**Published:** 2010-12-08

**Authors:** Salome Casutt-Meyer, Francesco Renzi, Mathias Schmaler, Naja J. Jann, Marlise Amstutz, Guy R. Cornelis

**Affiliations:** 1 Biozentrum der Universität Basel, Basel, Switzerland; 2 Departement Biomedizin, Universität Basel, Basel, Switzerland; Charité-University Medicine Berlin, Germany

## Abstract

*Yersinia* adhesin A (YadA) is an essential virulence factor for the food-borne pathogens *Yersinia enterocolitica* and *Yersinia pseudotuberculosis.* Suprisingly, it is a pseudogene in *Yersinia pestis*. Even more intriguing, the introduction of a functional *yadA* gene in *Y. pestis* EV76 was shown to correlate with a decrease in virulence in a mouse model. Here, we report that wild type (wt) *Y. enterocolitica* E40, as well as YadA-deprived E40 induced the synthesis of neutrophil extracellular traps (NETs) upon contact with neutrophils, but only YadA-expressing *Y. enterocolitica* adhered to NETs and were killed. As binding seemed to be a prerequisite for killing, we searched for YadA-binding substrates and detected the presence of collagen within NETs. E40 bacteria expressing V98D,N99A mutant YadA with a severely reduced ability to bind collagen were found to be more resistant to killing, suggesting that collagen binding contributes significantly to sensitivity to NETs. Wt *Y. pestis* EV76 were resistant to killing by NETs, while recombinant EV76 expressing YadA from either *Y. pseudotuberculosis* or *Y. enterocolitica* were sensitive to killing by NETs, outlining the importance of YadA for susceptibility to NET-dependent killing. Recombinant EV76 endowed with YadA from *Y. enterocolitica* were also less virulent for the mouse than wt EV76, as shown before. In addition, EV76 carrying wt YadA were less virulent for the mouse than EV76 expressing YadA_V98D,N99A_. The observation that YadA makes *Yersinia* sensitive to NETs provides an explanation as for why evolution selected for the inactivation of *yadA* in the flea-borne *Y. pestis* and clarifies an old enigma. Since YadA imposes the same cost to the food-borne *Yersinia* but was nevertheless conserved by evolution, this observation also illustrates the duality of some virulence functions.

## Introduction

The three pathogenic species of *Yersinia*, *Y. pestis*, *Y. pseudotuberculosis* and *Y. enterocolitica*, carry a 70-kb virulence plasmid, which encodes a type-III secretion (T3S) system consisting of the Ysc injectisome and the Yop effectors (for review [1]). Injection of the effectors is triggered by tight contact with a target cell [2], [3]. Macrophages, and presumably also polymorphonuclear neutrophils (PMNs), which are the main targets for T3S [4], engage *Yersinia* bacteria in the absence of any bacterial adhesin and, by doing so, trigger their fatal injection. In contrast, for other cell types, T3S only occurs in the presence of bacterial adhesins, which promote docking of the bacteria to the target cell [5], [6]. For *Y. enterocolitica*, the main adhesins playing this role are the *Yersinia* adhesin A (YadA), originally described as outer membrane protein 1 [7], [8], and the invasin (Inv) [5], [9], [10]. YadA is encoded by the same 70-kb virulence plasmid as the T3S system [7], [8] and its expression is co-regulated by the transcription activator VirF (LcrF) [11], [12], [13], which suggests that YadA and T3S act indeed cooperatively. In contrast to YadA, Inv is chromosomally encoded [14] and expressed only at low temperature [9], in agreement with an earlier role in pathogenesis (for review [15]).

YadA is a homotrimeric outer membrane protein forming a fibrillar matrix at the surface of *Y. enterocolitica* and *Y. pseudotuberculosis* [16]. It appears as lollipop structures covering the whole bacterial surface, made of a short C-terminal membrane anchor, a *ca* 18 nm long coiled-coil stem and a 5 nm long N-terminal globular head structure consisting of a left-handed parallel beta-roll [16], [17]. This structure makes YadA the archetype of a family of oligomeric coiled-coil adhesins (Oca) [16].

Model studies have proposed that trimerization involves not only the coiled-coil stem but also the C-terminal membrane anchor, which forms a 12-strand β-barrel from the four transmembrane β-strands of the three monomers. This β-barrel would form a pore-like structure through which the N-terminal head and coiled helical domains of the three monomer chains exit to the cell surface [18], [19]. The Oca family of proteins is thus viewed as a subset of autotransporters, the type Vc or trimeric autotransporters [20], [21]. The main difference with “conventional” (“type Va”) autotransporters is that N-terminal passenger domains are not cleaved off, but they function together at the bacterial surface to provide trivalent (high-avidity) ligands that can cluster receptors on eukaryotic cells [21].

YadA was first discovered because of its capacity to promote auto-agglutination of *Y. enterocolitica* and *Y. pseudotuberculosis* [7], [22], as well as adherence to many substrates including epithelial cells [23], [24], extracellular matrix [25], collagen [24], [25], [26], cellular but not plasma fibronectin [27], and laminin [28]. The very tight adherence to nucleated cells is mediated by the interaction between YadA and β1 integrin receptors but occurs indirectly through an integrin-extracellular matrix protein–YadA linkage [29], [30], [31] (for review [32]). YadA from *Y. pseudotuberculosis* – and to a much lesser extent also from *Y. enterocolitica* – leads to the entry into non-phagocytic cells [24], [30], [31], [33]. YadA might thus contribute, like Inv, to the entry of *Y. enterocolitica* into M cells, which leads to colonization of Peyer's patches but this is probably not its main role. Presumably of more relevance, at least for *Y. enterocolitica*, YadA confers resistance to the bactericidal activity of human serum [7], by binding factor H [34]. By preventing complement deposition, it also confers some degree of resistance to phagocytosis [35], [36]. Even more, YadA provides some resistance to killing by antimicrobial peptides (AMPs) from human granulocytes to *Y. enterocolitica* [37]. Because of all these properties, it is generally admitted that YadA is an important factor contributing to the pathogenesis of *Y. enterocolitica* gastroenteritis, which was actually shown in a mouse model [28].

It thus came as a surprise that *yadA* as well as *inv*, exist as pseudogenes in *Y. pestis* [22]. It has even been shown that inactivation of *yadA* and *inv* correlates with an increase of virulence [38], but this was contradicted later on [39].

PMNs, which are the first line of defence against invading microbial pathogens [40], [41], release granule proteins and chromatin that together form extracellular fibers called neutrophil extracellular traps (NETs) that bind and kill Gram-positive and Gram-negative bacteria [42], [43], [44], [45]. The mechanism for trapping by NETs has not yet been fully elucidated.

In this paper, we report that YadA confers a high level of sensitivity to killing by PMN-produced NETs to *Yersinia*, by mediating binding of the bacteria to NETs and thereby rendering them accessible to the AMPs present on the NETs. Thus, in the host, YadA has two antagonistic properties: it protects against complement killing and phagocytosis but, at the same time, it makes *Yersinia* more sensitive to NETs. This observation therefore shows that some factors can behave *in vivo* as two sided coins and it suggests an explanation as for why evolution selected for the loss of YadA in *Y. pestis*.

## Results

### YadA makes *Y. enterocolitica* E40 sensitive to NETs

**Figure 1:**
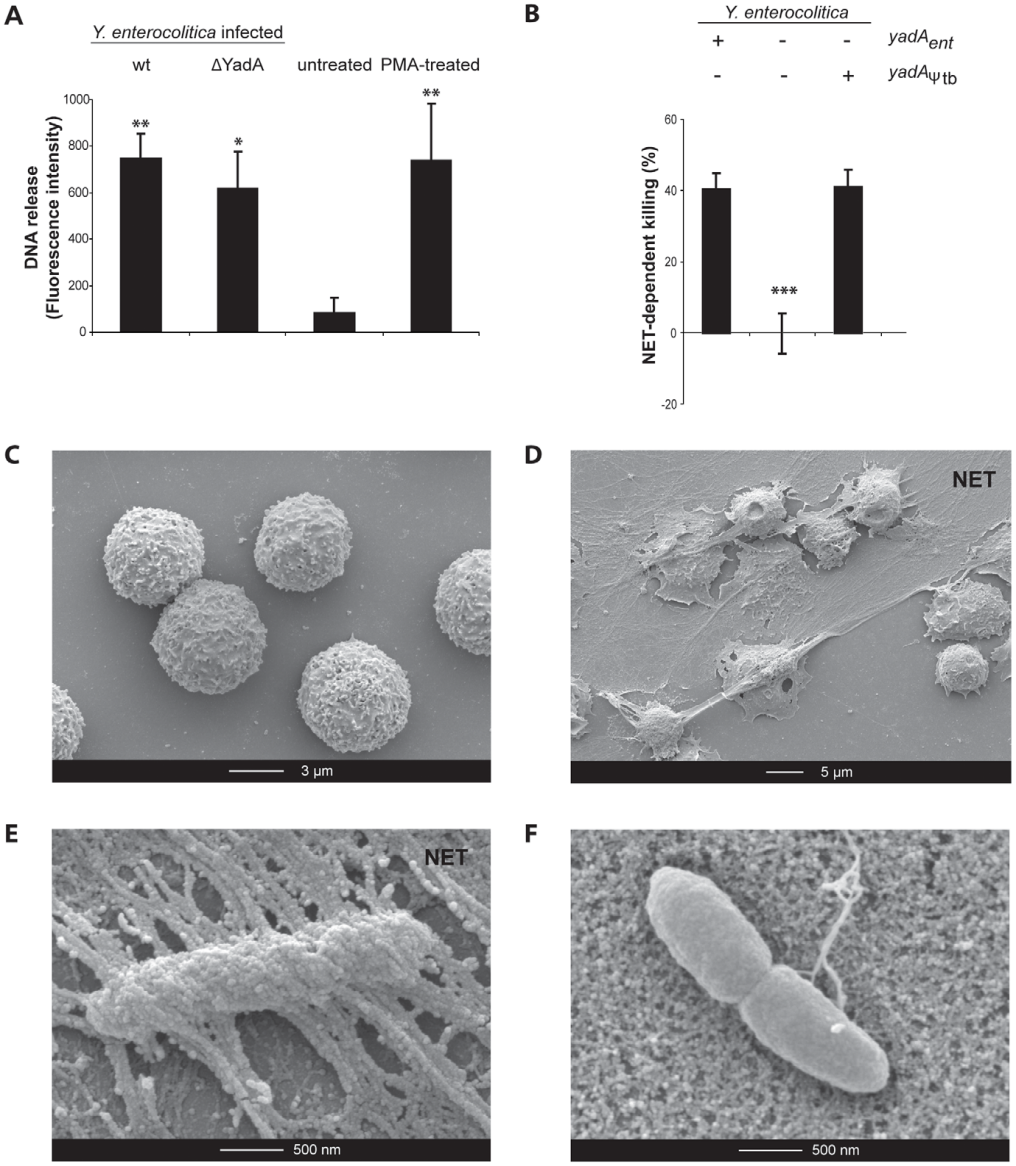
YadA renders *Y. enterocolitica* sensitive to NET-dependent killing. (**A**) *Y. enterocolitica* induces DNA release upon infection of PMNs. PMNs were infected for 120 min with *Y. enterocolitica* E40 (wt or ΔYadA) at an moi of 1. DNA release was quantified by Sytox staining. Untreated PMNs were used as negative control and NET formation was induced by PMA as positive control. Mean values from three or more experiments and standard deviation are shown including statistical significance in comparison to untreated PMNs with ** p<0.01 and * p<0.05 using one-way ANOVA. (**B**) % of *Y. enterocolitica* E40 (wt, ΔYadA and ΔYadA endowed with pSAM16 encoding YadA_Ψtb_) killed by PMA-triggered NETs (120 min infection at a moi of 1). Phagocytosis was prevented by the addition of Cytochalasin D (CytD). Mean values from three or more experiments and standard deviation are shown. Statistical significance is shown in comparison to *Y. enterocolitica* wt with *** p<0.001 using one-way ANOVA. (**C**) Scanning electron micrograph (SEM) of untreated human PMNs and (**D**) of NETs formed by human PMNs treated with PMA. (**E**) SEM of *Y. enterocolitica* E40 wt bacteria (expressing YadA) trapped in NETs after 120 min infection at an moi of 1. (**F**) *Y. enterocolitica* E40 ΔYadA bacteria induce NET formation but are not trapped (same conditions as in A) (SEM). NET structure covers the whole bottom.

After 120 min of infection with wild type (wt) *Y. enterocolitica* E40 bacteria at a multiplicity of infection (moi) of 1, human PMNs released as much DNA as when they were treated with phorbol myristate acetate (PMA), an artificial inducer of NETs, indicating that *Y. enterocolitica* E40 induce NET formation. NETs were visible by scanning electron microscopy (SEM), all around the bacteria. In agreement with this, ∼40% of bacteria were killed by PMA-induced NETs (**Fig. 1a–e**). *yadA* knockout *Y. enterocolitica* E40 (E40 ΔYadA) bacteria also induced the formation of NETs (**Fig 1a, f**), but in contrast to wt bacteria, they were neither trapped (**Fig 1f**), nor killed (**Fig 1b**). This experiment revealed, for the first time, that YadA has some negative effect for the pathogen.

### Collagen binding contributes to sensitivity to NETs

**Figure 2:**
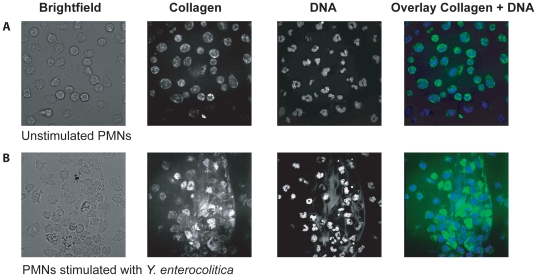
NETs contain collagen. (**A**) Untreated PMNs. DNA (blue) and collagen (green) visualized by immunofluorescence. (**B**) Same staining after the PMNs have been infected with *Y. enterocolitica* E40 for 120 min at an moi of 1. Smears represent NETs. 60 x magnification, DNA (blue), collagen (green) and brightfield.

**Figure 3:**
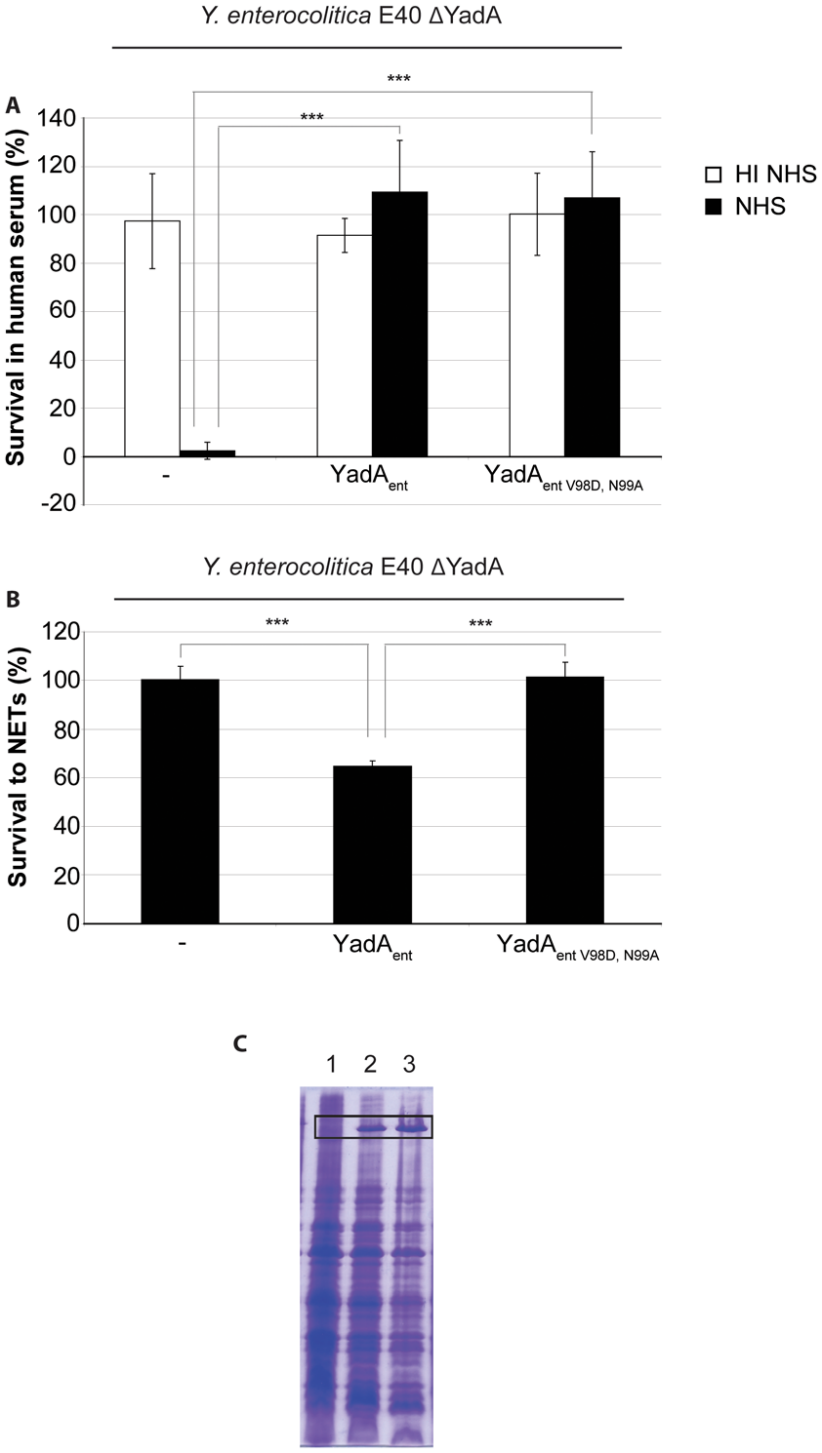
Collagen binding contributes to the sensitivity of *Y. enterocolitica* to NETs. (**A**) Mutation of the collagen binding motif has no effect on serum resistance. % survival of *Y. enterocolitica* E40 ΔYadA, E40 ΔYadA (pFR1) expressing YadA_ent_, or E40 ΔYadA (pFR2) expressing YadA_entV98D,N99A_, incubated for 1 hour in the presence of 10% normal human serum (NHS) or heat-inactivated human serum (HI NHS). (**B**) % Survival to NET-dependent killing of *Y. enterocolitica* E40 ΔYadA, E40 ΔYadA (pMA1) expressing YadA_ent_, or E40 ΔYadA (pSAM23) expressing YadA_entV98D,N99A_. (**C**) YadA_entV98D,N99A_ forms trimers. SDS PAGE analysis of total cells from *Y*. *enterocolitica* E40 ΔYadA, E40 ΔYadA (pFR1) expressing YadA_ent_, or E40 ΔYadA (pFR2) expressing YadA_entV98D,N99A_. The band corresponding to trimeric YadA is boxed.

Since the scanning electron micrographs suggested that sensitivity to NETs correlates with attachment, we investigated whether NETs contain substrates to which YadA is known to bind. We indeed found that NETs contain collagen (**Fig. 2**), and we sought to test whether collagen was responsible for the attachment of YadA to NETs. To this end, we took advantage of the structural analysis of type-I collagen binding to the trimeric head of YadA [17]. This study established that substitution of surface-exposed residues 98 and 99 drastically reduces collagen binding without affecting the global structure [17]. We complemented E40 ΔYadA with a plasmid-borne *Y. enterocolitica yadA* gene (called *yadA_ent_*), and checked that the recombinant E40 was sensitive to NET-mediated killing (**Fig 3b**). We then introduced the V98D and N99A substitutions and tested the sensitivity of E40 expressing YadA_entV98D,N99A_ to NET-mediated killing. As shown in **Fig 3b**, the two substitutions significantly affected killing by NETs but the substitutions did not affect trimerization (**Fig 3c**) and serum resistance (**Fig 3a**). We conclude from this that collagen-binding significantly contributes to sensitivity to NET-dependent killing, most likely by promoting attachment of *Y. enterocolitica* to NETs.

### YadA makes *Y. pestis* sensitive to NET-dependent killing

**Figure 4:**
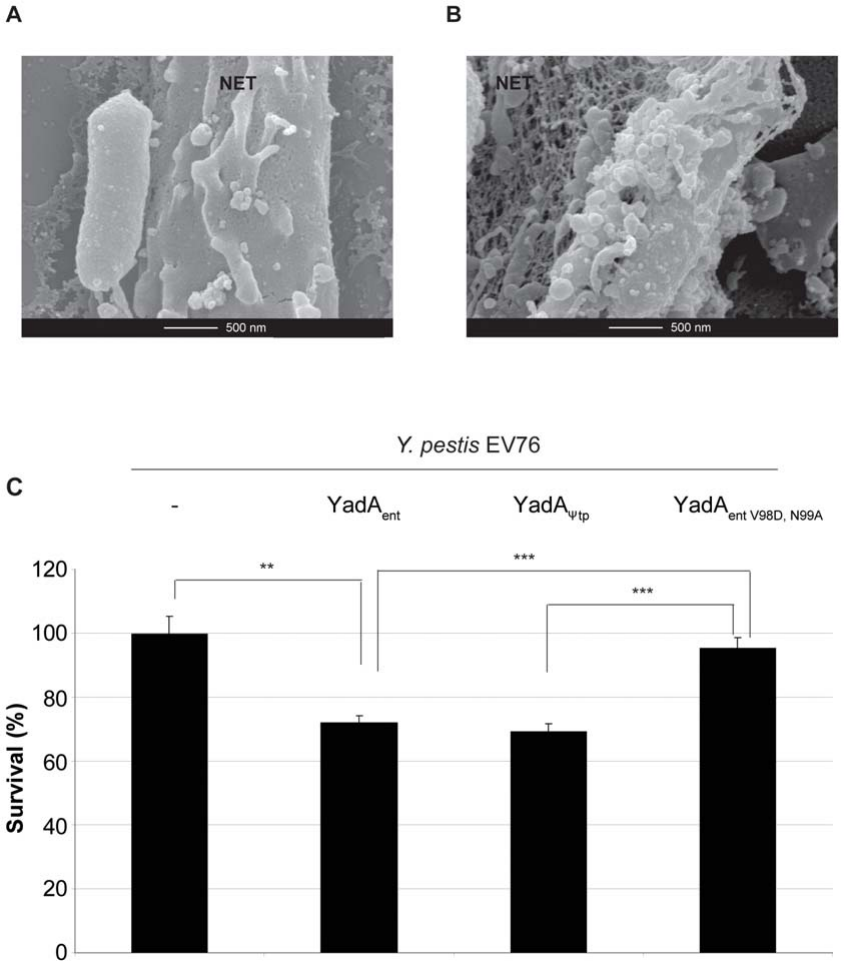
YadA renders *Y. pestis* EV76 sensitive to NET-dependent killing. (**A**) *Y. pestis* EV76 wt bacteria (do not express YadA) induce NET formation but are not trapped (SEM, same conditions as in Fig 1). (**B**) *Y. pestis* EV76(pMA1) expressing YadA_ent_ trapped in NETs (same conditions as in A). (**C**) % of survival of *Y. pestis* EV76 wt, EV76(pMA1) endowed with YadA_ent_, EV76(pSAM23) endowed with YadA_entV98D,N99A_ or EV76(pSAM16) endowed with YadA_Ψtb_ in the presence of PMA-triggered NETs. Phagocytosis was prevented by the addition of CytD. Mean values from three or more experiments and standard deviation are shown. Statistical significance is shown with ** p<0.01 and *** p<0.001 using one-way ANOVA.

To test whether this negative effect could help explain why *yadA* is a pseudogene in *Y. pestis*, we first set out to test if *Y. pestis* EV76 wt was resistant to NET-dependent killing. Consistently with our *Y. enterocolitica* data, wt *Y. pestis* EV76 devoid of YadA did induce NET formation, but were neither trapped in NETs, nor killed (**Fig 4a, c**). In contrast, *Y. pestis* EV76 expressing YadA from *Y. enterocolitica* (YadA_ent_) did not only induce NET formation, but were also trapped and killed by NETs (**Fig. 4b, c**). We then expressed YadA from a *Y. pseudotuberculosis* strain (YadA_Ψtb_) (94% similar to frame-shift corrected YadA from *Y. pestis* EV76) in ΔYadA *Y. enterocolitica* and found that indeed, ΔYadA *Y. enterocolitica* E40 bacteria expressing YadA_Ψtb_ were sensitive to NETs (**Fig. 1b**). *Y. pestis* EV76 expressing YadA_Ψtb_ also became sensitive to NET-dependent killing (**Fig 4c**). In contrast, expression of the YadA collagen binding mutant YadA_entV98D,N99A_ did not confer a significant sensitivity (**Fig 4c**).

### YadA reduces the virulence of *Y. pestis* and collagen-binding contributes to this effect

**Figure 5:**
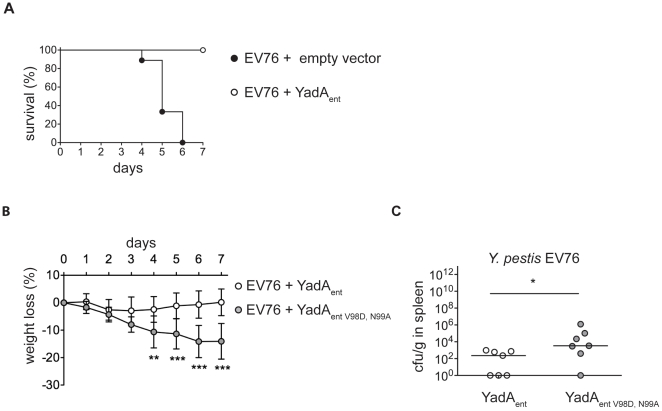
YadA drastically reduces the virulence of *Y. pestis* EV76 for mice. Collagen-binding contributes to this effect. Iron loaded Balb/c mice were infected i.p. with 5×10^3^ CFU of *Y. pestis* EV76(pACYC184) (empty vector), EV76(pFR1) (YadA_ent_) or EV76(pFR2) (YadA_entV98D,N99A_). (A) Survival, (B) weight loss, and (C) bacterial load in spleens of mice on day 7. Data are represented as mean ± standard deviation for weight loss and median for CFU/g of at least 7 mice per group in two independent experiments. Significant differences are indicated by * p<0.05, ** p<0.01, and *** p<0.001.

In order to determine whether the observations on sensitivity to NETs had some *in vivo* relevance, mice were inoculated with *Y. pestis* EV76 or EV76 containing pACYC184 (medium-copy) derivatives expressing YadA_ent_ or YadA_entV98D,N99A_ from the native *yadA* promoter. Mice were also administered chloramphenicol in their drinking water to guarantee plasmid maintenance and were monitored for 7 days after infection. All mice infected with *Y. pestis* EV76 containing the empty vector died before day 6. They had a weight loss of 20–30% and high bacterial load in the spleen (median, 25th and 75th percentile, 4.8×10^7^, 8.0×10^6^ and 1.7×10^8^ CFU/g spleen) at the time of death. The mice infected with *Y. pestis* EV76 expressing YadA_ent_ had no significant weight loss and survived until day 7, when they were sacrificed (**Fig 5a**). These data are in perfect agreement with the initial report of the Wolf-Watz laboratory that YadA reduces virulence of *Y. pestis* EV76 [38]. No mice inoculated with *Y. pestis* EV76 expressing YadA_entV98D,N99A_ died after infection (data not shown) but they had a significant weight loss (**Fig 5b**). Bacterial burden in spleens (similar weight p = 0.0728) was also higher in mice infected with *Y. pestis* EV76 expressing YadA_entV98D,N99A_ than in mice infected with *Y. pestis* EV76 expressing YadA_ent_ (**Fig 5c**). These data show that the collagen-binding mutation reduced the cost of YadA on virulence although it did not abolish it.

## Discussion

The experiments presented here demonstrate that YadA makes *Y. enterocolitica* and *Y. pestis* sensitive to killing by NETs. This sensitivity results from YadA-mediated adherence to NETs. Adherence is likely to be multifactorial since NETs have many constituents [46] and YadA is known to adhere to many substrates [32]. Nevertheless, collagen presumably plays an important role in trapping *Y. enterocolitica* and *Y. pestis* in NETs. Indeed, *Y. enterocolitica* E40 and *Y. pestis* EV76 expressing a variant of YadA that binds less to collagen were also significantly less sensitive to NET-mediated killing. Thus, our data show that YadA and, more specifically, collagen-binding imposes a fitness cost to *Y. enterocolitica* and *Y. pestis*.

Interestingly enough, collagen binding was shown to be essential for the virulence of *Y. enterocolitica* in mice inoculated intragastrically [28]. Hence, this explains why YadA was conserved in the food-borne *Y. enterocolitica* and *Y. pseudotuberculosis*, the direct ancestor of *Y. pestis* [47], in spite of the fitness cost. The situation appeared to be different for *Y. pestis*, usually inoculated subcutaneously by a flea bite. Indeed, our data confirm the old but controversial observation that YadA reduces the virulence of *Y. pestis* for the mouse [38]. The finding that YadA confers sensitivity to killing by NETs may thus explain this old observation and why YadA is a pseudogene in *Y. pestis*. The mice experiments also showed that collagen binding contributes to the YadA-mediated reduction of virulence. However, a mutant YadA known to have a collagen-binding ability reduced to 4% of wt [17], did still reduce virulence for the mouse. This indicates that either a very low level of collagen binding suffices to confer sensitivity to NETs *in vivo*, or that YadA has still other adverse effect(s) *in vivo*, than conferring NET sensitivity. Thus, our data confirm the controversed report that YadA has a strong fitness cost for *Y. pestis* EV76 in the iron-loaded mouse model [38] and they suggest that this results from NET-dependent killing. They also show that collagen binding contributes to this adverse effect.

It is difficult to argue why YadA was lost in *Y. pestis* and kept in its ancestor *Y. pseudotuberculosis* and in *Y. enterocolitica*. Since *Y. pestis* adapted to intra-dermal transmission by flea bites while the two others enter their host via the oral route, it seems logical to speculate that YadA plays an essential role in the early stages of the oral infection but no such role was demonstrated so far. The adaptation to a new transmission route (the flea) would thus have made YadA dispensible for *Y. pestis*. However, any pathogen breaking the skin or mucosal barrier must be able to resist the bactericidal activity of complement. Not surprisingly, *Y. pestis* has acquired an alternative complement resistance mechanism [48] and this event could have been a prerequisite for the loss of YadA.

To conclude, this study shows that virulence factors can be double-edged swords, illustrating the complexity of the host-pathogen interactions.

## Materials and Methods

### Ethics statement

Animal experiments were performed in strict accordance with institutional and guidelines of the Swiss veterinary law (article 13a TSchG; 60–62 TSchV). The protocol was reviewed and approved by the veterinary office of the canton Basel (Permit Number: 1397-Inflammation and mouse peritonitis model in mice, valid until 2010-12-31).

Human blood samples for this study were provided by the “Blutspendezentrum SRK beider Basel”, from healthy volunteers after obtaining written informed consent, in agreement with the guidelines of the “Ethikkommission beider Basel EKBB”.

### Bacterial strains and media

Bacterial strains are listed in **Table 1**. *Y. enterocolitica* and *Y. pestis* were grown as in ref [36]. When needed, YadA expression was induced with 0.2% arabinose for 60 min at 37°C before infection of PMNs.

**Table 1:** Bacterial strains and plasmids used in this study.

Bacterial strains	Genotype	References
*Y. enterocolitica* E40	Serotype O:9, wt, (pYV40)	[54]
*Y. enterocolitica ΔYadA*	E40 (pLJM4029) *yadA::pLJM31*, SmR	[3]
*Y. enterocolitica* W227	Serotype O:9, wt, (pYV227)	[55]
*Y. pestis EV76*	*pla*+, *caf*1+, (pCD1), Δ*pgm*	[38]
*Y. pseudotuberculosis*	Serotype 1B wt, isolated from a hare	gift of G. Wauters (13215/7)

Expression vectors	Genotype	References
pACYC184	Ori p15A, cml^R^,tet^R^ 4.2 kb	[56]
pBAD	P*araBAD*, Amp^R^, Myc and HisA tags	Invitrogen

Expression plasmids	Construction	References
pMA1	*yadA* from *Y. enterocolitica* W227 (*yadA_ent_*) cloned into *Nco*I and *Eco*RI sites of pBAD after amplification with primers 5115 and 4561. Ara^I^, Amp^R^. Expression from the pBAD promoter.	This study
pSAM16	Full-length *yadA* from *Y. pseudotuberculosis*, (*yadA_Ψtb_*) cloned into *Nco*I and *Eco*RI sites of pBAD after amplification with primers 5113 and 5114. Ara^I^, Amp^R^Expression from the pBAD promoter.	This study
pSAM23	pMA1 *yadA_entV98D_*,*_N99A_*Substitution using primers 5491 and 5492.Expression from the pBAD promoter.	This study
pLJM32	pBBRMCS2 expressing full-length *yadA_ent_* from its native promoter. Km^R^	[3]
pFR1	*yadA_ent_* with its native promoter from pLJM32 cloned in *Hin*dIII-*Xba*I sites of pACYC184, Cm^R^	This study
pFR2	*yadA_ent_*_ V98D,N99A_ with the *yadA_ent_* promoter cloned inin *Hin*dIII-*Xba*I sites of pACYC184, Cm^R^	This study

**Site-directed mutagenesis** was performed by PCR at sites indicated in ref [49]. Primers are given in **Table 2**.

**Table 2:** Oligonucleotides used in this study.

Code	Sequence
4561	**GGAATTCTTACCACTCGATATTAAATGATGCAT**
5113	GACCATGGCCACTAAAGATTTTAAGATCAGTGTCTC
5114	GGAATTCTTACCACTCGATATTAAATGATGCGT
5115	**GACCATGGCCACTAAAGATTTTAAGATCAGTGTCTC**
**5491**	**AACAGGCGATGCTTCTGTTGCAATTGGTCCTTTA**
**5492**	**GCAACAGAAGCATCGCCTGTTGCAATTGAACCAGC**

**Human PMNs** were isolated from human blood using the Dextran-Percoll protocol, adapted with modifications from ref [50].

***In vitro*** **analysis of NET-dependent killing** was adapted from ref [51]. Briefly, freshly isolated human PMNs were resuspended at 10^6^ ml^−1^ in D-PBS (Gibco) supplemented with 2% Ab-depleted pooled human serum (Scipac Ltd.). To induce NET formation, PMNs were incubated for 30 min with 25 nM PMA (Sigma) at 37°C and 5% CO_2_. Cyt. D (10 µg/ml, Sigma) was added for 20 min before infection to block phagocytosis. Bacteria were added at an moi of 1 to PMNs. To determine killing, samples were taken at 120 min after infection, diluted in ddH_2_O and plated. As control for NET-dependent killing, NETs were degraded by incubation with 50 U/ml of RNase-free and protease-free DNase-1 (Worthington) prior to the addition of bacteria (data not shown). To determine reference values, an equal number of bacteria was incubated without PMNs. The survival rate was calculated by reference to the sample without PMNs.

**Quantification of DNA released by activated neutrophils** was adapted from ref [52]. Freshly isolated human PMNs were resuspended in D-PBS (Gibco) supplemented with 2% Ab-depleted pooled human serum (Scipac Ltd.). Cells were seeded into 96-well microtiter plates (2×10^5^ cells/well, Falcon) and either stimulated with PMA (25 nM, Sigma) for 30 min, or infected with bacteria at an moi of 1 for 120 min at 37°C and 5% CO_2_ in a humidified incubator. If indicated, cells were left untreated as control. After incubation, Sytox green (10 µM, Molecular Probes), a cell impermeant DNA binding dye, was added to the cells to detect extracellular DNA. Fluorescence was measured at 485 nm with a Wallac Victor^2^ 1420 Multilabel counter (Perkin Elmer).

**Scanning electron microscopy** was adapted from ref [52]. Freshly isolated human PMNs were resuspended in D-PBS (Gibco) supplemented with 2% Ab-depletet HI NHS (Scipac Ltd.). Cells were seeded on 12 mm 0.001% poly-D-lysine coated coverslips in 24-well microtiter plates (10^6^ cells/well, Falcon). The medium was replaced with hRPMI and bacteria were added at an moi of 1. After 2 h of infection at 37°C and 5% CO_2_, samples were fixed with 2.5% glutaraldehyde and dehydrated with graded ethanol series (30%, 50%, 70%, and 100%). After dehydration and critical-point drying, the specimens were coated with 2 nm platinum-film and analyzed on a Philips XL-30 ESEM scanning electron microscope at the ZMB, Biozentrum, University of Basel.

### Collagen detection

Freshly isolated human PMNs were resuspended in RPMI 1640 (Gibco) supplemented with 2% antibody-depleted pooled human serum (Scipac Ltd.) and 2% L-glutamine (Gibco) and seeded on Falcon™ culture slides (Becton Dickinson) coated with 0.001% poly-D-lysine (5×10^5^ cells/well). PMNs were infected for 120 min with *Y. enterocolitica* wt bacteria at an moi of 1, washed with D-PBS (Gibco), fixed with 4% paraformaldehyde for 60 min at 37°C and then blocked overnight at 4°C with D-PBS containing 3% bovine serum albumine. Collagen was labelled with mouse anti-human collagen type I antibody (1∶1000; Sigma). FITC-conjugated secondary antibody (1∶200; Southern Biotech) and Hoechst DNA staining dye (1∶10000; Sigma) were added and slides were incubated for 30 min at RT, washed 4 times with D-PBS, mounted with antifade reagent (Vector Laboratories) and analyzed on an Olympus IX81F-3 microscope mounted with a high speed Yokogawa spinning head at 60× magnification.

**Serum sensitivity** was adapted from ref [7]. Bacterial cultures were grown overnight in BHI at 28°C in an orbital shaker at 150 rpm. Cultures were then diluted in BHI to OD_600_ = 0.1, grown for 2 hours at 28°C and then shifted to 37°C for 3 hours to induce YadA expression. Bacterial cultures were then suspended in 0.5 ml PBS at a cell density of 2×10^7^ cells/ml and incubated for 1 hour at 37°C in the presence of 10% (vol\vol) fresh or heat inactivated human serum. Viable counts were determined by plating appropriate dilutions onto LB plates.

### Mice infections

Wild-type inbred Balb/c mice were bred and maintained under specific pathogen-free conditions in the Animal House of the Department of Biomedicine, University Hospital Basel. In order to favor plasmid maintenance in *Y. pestis* strains, mice were administered chloramphenicol in their drinking water (0.125 mg/ml) from 48 hours before infection until the end of the experiment. Since *Y. pestis* EV76 has a 102-kb deletion of the *pgm* locus encoding synthesis and capture of the siderophore yersiniabactin [53], 2.5 mg iron dextran (Serumwerk Bernburg AG, Germany) in 0.9% NaCl (Bichsel AG, Switzerland) were injected into the peritoneum of mice three hours before inoculation. Bacteria were grown overnight in BHI at 28°C in an orbital shaker at 150 rpm. Cultures were then diluted in BHI to OD_600_ = 0.1 and grown for 3 hours at 28°C and then shifted to 37°C for 1.5 hours. Bacterial cultures were diluted to 1×10^4^ CFU/ml in 0.9% NaCl containing 1% Desferal (Novartis, Switzerland). Mice (6–7 weeks old, females) were inoculated i.p. with 0.5 ml (5×10^3^ CFU) of *Y. pestis* EV76(pACYC184), *Y. pestis* EV76(pFR1) or *Y. pestis* EV76(pFR2) suspensions. Weight loss and survival of mice was monitored during infection. Either at d7 after infection or at terminal stage of the infection, mice were killed by CO_2_ asphyxiation, spleens were removed and homogenized to determine bacterial load. Homogenized spleens were serially diluted in 0.9% NaCl (Bichsel AG, Switzerland) and plated on LB agar plates to determine bacterial load. To determine plasmid stability colonies were replica plated on LB agar plates with and without 10 µg/ml chloramphenicol. If plasmid stability of remaining bacteria was higher than 83%, mice were used for analysis. In Kaplan-Meier plots, log rank test was used to compare survival among infected mice. 2-way ANOVA test was used for statistical analyses of weight loss of mice after infection. CFU in organs were analyzed with Mann-Whitney. Statistical analysis was done with Prism 5.0c (GraphPad Software, Inc.). A p-value of p<0.05 was considered statistically significant.
